# Can a Light Detection and Ranging (LiDAR) and Multispectral Sensor Discriminate Canopy Structure Changes Due to Pruning in Olive Growing? A Field Experimentation

**DOI:** 10.3390/s24247894

**Published:** 2024-12-10

**Authors:** Carolina Perna, Andrea Pagliai, Daniele Sarri, Riccardo Lisci, Marco Vieri

**Affiliations:** Department of Agricultural, Alimentary, Environmental and Forestry Sciences, Biosystem Engineering Division—DAGRI, University of Florence, Piazzale delle Cascine 15, 50144 Florence, Italy; carolina.perna@unifi.it (C.P.); andrea.pagliai@unifi.it (A.P.); riccardo.lisci@unifi.it (R.L.); marco.vieri@unifi.it (M.V.)

**Keywords:** proximal sensing, olive tree, pruning management, LiDAR, multispectral sensor, ground-vehicle

## Abstract

The present research aimed to evaluate whether two sensors, optical and laser, could highlight the change in olive trees’ canopy structure due to pruning. Therefore, two proximal sensors were mounted on a ground vehicle (Kubota B2420 tractor): a multispectral sensor (OptRx ACS 430 AgLeader) and a 2D LiDAR sensor (Sick TIM 561). The multispectral sensor was used to evaluate the potential effect of biomass variability before pruning on sensor response. The 2D LiDAR was used to assess its ability to discriminate volume before and after pruning. Data were collected in a traditional olive grove located in Tenute di Cesa Farm, in the east of Tuscany, Italy, characterized by a 4x6 m planting layout and by developed plants. LiDAR data were used to measure canopy volumes, height, and diameter, and the generated point cloud was studied to assess the difference in density between treatments. Ten plants were selected for the study. To validate the LiDAR results, manual measurements of the canopy height and diameter dimensions of the plants were taken. The pruning weights of the monitored plants were obtained to assess the correlation with the canopy characterization data. The results obtained showed that pruning did not affect the results of the multispectral sensor, and the potential variation in canopy density and porosity did not lead to different results with this instrument. Plant volumes, height, and diameters calculated with the LiDAR sensor correlated well with the values of manual measurements, while volume differences between before and after pruning obtained good correlations with pruning weights (Pearson correlation coefficient: 0.66–0.83). The study of point cloud density in canopy thickness and height showed different shapes before and after pruning, especially in the former case. Correlations between point cloud density obtained from LiDAR and multispectral sensor results were not statistically significant. Even if more studies are necessary, the results obtained can be of interest in pruning management.

## 1. Introduction

The global olive industry faces increasing pressures to enhance productivity, optimize resource use, and ensure sustainability in the context of climate change [1,2,3]. As a result, the olive-growing sector is required to change and adapt its practices to reach the objectives of both sustainability and productivity [4,5]. To face and oppose the negative effects of climate change, for example, the farmers need to choose between multiple adaptation strategies ranging from long-term strategies (clone selection, relocation) to short-term strategies (irrigation and soil management, pruning) [6,7]. It is critical to analyze and evaluate the best strategies to adapt and overcome those challenges and limitations, with a particular focus on the increase in the efficiency of existing systems, particularly through innovation [8,9]. In this context, data and data collection for management and decision-making are fundamental strategies. Collecting data and information allows insight into multiple aspects of the olive orchard, physiologically and technically, that can be fundamental in managing challenging situations [10,11]. Information on the physiological condition of the trees, production quality and potential yield, and the vegetative–productive balance is particularly impactful for olive tree management.

Precision agriculture (PA) has emerged as a key strategy in this regard, leveraging technological innovations to enable more targeted and effective management practices, particularly in high-value crops like olives [12]. Precision agriculture uses advanced technologies, such as remote sensing, machine learning, and geospatial analysis, to monitor and manage crop growth at a fine scale. In the context of olive cultivation, PA has shown significant potential to optimize various aspects of the production process, including irrigation management, pest control, vegetative and nutritional state, and harvesting operations [13,14,15,16,17]. For instance, PA technologies can help monitor soil moisture levels and plant health, enabling more efficient use of water and agrochemicals. These are crucial in regions where olive cultivation is prominent and resources are scarce [18,19]. Another element to be considered in this context is crop modeling. Crop modeling through data collected by PA is a valuable tool that provides growers with reliable information on crop status during the season and can assist in decision-making for implementing appropriate management practices [20,21]. One of the key challenges in implementing precision agriculture in olive orchards is the need for reliable and timely data collection. Traditionally, olive management has relied on manual inspections and sampling, which are labor-intensive and error-prone. However, the advent of automated data collection systems, such as ground-mounted LiDAR (light detection and ranging) and multispectral sensors, has the potential to revolutionize how data are gathered and utilized in olive management [17]. LiDAR is an optical sensor technology which functions based on the emission of intense, focused beams of laser energy. LiDAR tools can then measure the time it takes for reflections of the laser from a target to be detected by the sensor [22]. Specifically, the operativity of LiDAR technology is based on an “immediate” principle: knowing that the propagation speed of light is fixed (ca. 300,000 km s^−1^), it is possible to calculate the time it takes for a light beam to travel from a source to a (reflecting) target and back to the light detector. Through the detection of the distance between the sensor and the target, a LIDAR system generates an accurate 2D or 3D map of its surroundings. Thanks to the possibility of reconstructing an element in 3D, the LiDAR sensor can be used in olive management and data collection for detecting plant volume, geometries, and LAI (leaf area index) [23,24,25,26]. Consequently, these sensors provide detailed information about the structure of olive trees, which can be used to make more informed management decisions [27,28,29]. In particular, LiDAR can generate information that can potentially be used for crop canopy modeling [30,31], precise spraying [32], and for studying the canopy dynamic in pruning operations [33]. However, LiDAR data can be difficult to manage, with different algorithms (voxelization, α-shape, DBSCAN algorithm, and others) and software necessary for the digitalization of the point obtained and for the estimation of the parameters desired [22]. Multispectral sensors, usually mounted on unmanned aerial vehicles (UAVs), can be profitable in managing the canopy development characterization and estimation, particularly in obtaining data that can be useful in managing operations like fertilization or pruning. By studying the canopy’s vegetative status, it is possible to obtain information of interest in the evaluation of different forms of management: in particular, by knowing the vegetative development of the plants, it is possible to apply a different dose of fertilizer based on the actual need of the crop, as well as this information can be used to evaluate a different pruning management and the quantity of wood removed to maintain the correct vegetative–productive equilibrium [29,34]. On the downside, the application of precision farming, digitalization, and data collection in olive management presents constraints and problems, particularly due to the complexity of the use and applications of those new technologies, particularly in the data collection, elaboration, and analysis phases [35] which can be too complex to manage without the proper knowledge [36].

Pruning is a key phase of olive orchard management, playing a fundamental role in shaping tree architecture, regulating canopy density, and optimizing light penetration throughout the tree [37]. These factors are crucial for ensuring health and productivity, as they directly influence photosynthesis, fruit set, and overall yield quality. By controlling the tree’s structure, pruning helps maintain a balance between vegetative growth and fruit production [38,39,40], minimizes disease incidence by improving air circulation, and enhances the efficiency of mechanical harvesting [41,42]. Thus, effective pruning practices are essential for maximizing olive production and sustaining the long-term health and productivity of the orchard [37,43,44]. Nonetheless, the effectivity for pruning can vary depending on the systems and planting layout used: traditional (250–300 trees per hectare with planting layouts of 6 × 5 m, 6 × 7 m, and 7 × 7 m), intensive (layout of 5 × 5 m and 2.5 × 5 m, with 400–600 trees per hectare), and super intensive (1000 trees per hectare, the spacing is usually 4 m within the row and 1.3–1.5 m in the row) systems require different management, considering the different plant development and light interception [27,43,45]. Using sensors and data collection can reduce the gaps and the difficulties due to different layouts and management and can improve the management of the orchards in different contexts.

This research aimed to assess whether two sensors, optical and laser, evidenced the change in canopy structure due to the pruning. Specifically, the first was a multispectral sensor, and the second was a 2D LiDAR sensor. The objectives were the following:

1. Evaluate whether the variation in the canopy density due to pruning entails a variation in the results of the multispectral sensor. The aim was to assess whether pruning resulted in a change in biomass such that the multispectral sensor responded differently. For this research, sensors mounted on a ground vehicle were preferred. This is because a sensor positioned from a top view (like in the cases of satellite images or unmanned aerial vehicle images) may not take into account the vertical component of the plant but only look at it from above [46];
2. Assess if the LiDAR sensor can obtain the plant’s canopy’s physical characteristics and evaluate the LiDAR sensor’s capability to highlight differences between before and after pruning through different computational methods. This could allow the prediction of the wood weight removed and, in general, could give information of interest in optimizing the pruning management phase;
3. Investigate the relationship that the results obtained from the two sensors might have with each other to evaluate their joint use in decision-making, in particular by studying the interactions between the multispectral sensor and LiDAR results, assessing their relationship, and the potentially greater information that can be obtained from these interactions of managing the pruning practice, considering the possibility to use the information obtained to structure a more balanced pruning.

## 2. Materials and Methods

### 2.1. Experimental Site

The experiment was conducted at the Ente Terre Regionali Toscane Centre for Innovation Testing and Transfer located on the Tenuta di Cesa Farm in Marciano della Chiana, Arezzo, central-west Tuscany, Italy (coordinates: 43°19′9″ N, 11°47′51″ E, altitude 328.312 m AMSL).

The climate of this area is classified as Csa under the Köppen and Geiger systems, indicating a Mediterranean climate characterized by temperate and dry conditions with hot summers. The coldest month’s average temperature is above 0 °C, with at least one month having an average temperature above 22 °C and at least four months with an average temperature exceeding 10 °C. The mean annual temperature is 13 °C, and the average yearly precipitation is 871 mm (according to Consorzio LaMMA c/o CNR-IBE Florence research area).

The soils belong primarily to USDA Hydrologic Group C, which means they have a moderately high runoff potential when fully saturated, and water transmission through the soil is limited. A 2019 soil survey using electrical resistivity identified zones with similar resistivity, leading to site-specific soil sampling and analysis for each zone. The experimental site’s soil showed limited variability in electrical resistivity with the following characteristics: medium clay content (41%), lower silt and sand content (33% and 26%, respectively), and a moderately alkaline pH (8.1).

An olive orchard spanning 1.95 ha was chosen for the experiment. This orchard is designed as a germplasm trial orchard and includes five different cultivars: Pendolino, Colombino, Scarlinese, San Francesco, and Piangente. The soil was not tilled, and the farm only mowed the grass cover regularly, leaving the soil with a perennial cover. The olive trees were planted in June 2009 with a 5 × 6 m planting layout, oriented north-south, and managed using a multi-branched polyconic vase pruning system, with an average of three primary branches. The plants presented an average height of 3.5 m, and the canopies stood 0.7 m from the ground. The plants had the three main branches arranged at an angle of about 105–115 degrees to the ground. The canopy was managed by the farm in such a way as to limit the overlapping of neighboring canopies to ensure the proper penetration of light between the plants. In managing the canopy, it was preferred to enable the development of the branches towards the inter-row to try to limit, as mentioned above, the crossing of branches of different plants. Although a drip irrigation system is available, it was not used during the experiment.

The case study was carried out on 16 March 2023, during the pruning period in Tuscany. Sensor data were collected before and after the pruning. Ten plants were selected from the olive orchard based on their different development and wood structure; the difference was assessed visually. The different development was due to the intrinsic characteristics of the various cultivars: the plants’ cultivars were San Francesco (high vigor, assurgent habit, low crown density), Piangente (medium vigor, pendulous habit, high crown density), and Scarlinese (medium vigor, assurgent habit, high crown density) [47]. The first data collection with the sensors, before the pruning, was carried out at noon, and the second data collection was carried out one hour later, at 1 p.m., to allow the pruning of the plants and the measurement of the pruning weight. 

### 2.2. Multispectral and LiDAR Measurements

For canopy and vegetation index (VI) characterization, the OptRx^®^ ACS 430 crop sensor (AgLeader Technologies, Ames, IA, USA) was used. The OptRx is an active sensor that emits a light beam that strikes the plants’ canopies and measures the reflected light. It records reflectance in the 630–685 nm (red), 695–750 nm (red edge), and 760–850 nm (near-infrared) wavebands. The sensor stores these canopy response wavelengths as data points and automatically calculates the NDVI and NDRE indices using these wavebands. Each data point includes information on the values of the three wavelengths and the corresponding vegetation indices derived from the red, red edge (RE), and near-infrared (NIR) wavebands using Formulas (1) and (2), written as follows:

NDVI = NIR − RED/NIR + RED(1)

NDRE = NIR − RE/NIR + RE(2)

The multispectral sensor was mounted on a Kubota B2420 tractor (Kubota, Osaka, Japan) to automate data collection. This sensor was paired with a GNSS receiver, the GPS 6500 from AgLeader Technology (Ames, IA, USA), which was installed on the tractor’s frontal roll-over protective structure (ROPS) to provide georeferencing for each point collected by the OptRX sensor in the WGS85 reference system.

Terrestrial LiDAR was used to obtain the point cloud necessary for the 3D canopy reconstruction of the selected plants. The LiDAR used was the 2D Lidar TIM 561 (Sick, Waldkirch, Germany), and it was implemented as a mobile laser scanner (MLS). It was mounted on the rear part of the Kubota B2420 tractor with a staff firmly connected to the rear ROPS. This way, the LiDAR was positioned at 1.95 m from the ground, a height necessary for reaching the uppermost part of the canopies (average of 3.5 m). This LiDAR has an angular resolution of 0.33°, a working range from 0.05 m to 10 m, a scanning angle of 270°, and a scanning frequency of 15 Hz.

The data collection setup included a Panasonic ToughPad FG-Z1 (Panasonic Corporation, Kadoma, Japan) for hardware integration and data storage. All points data were saved in .csv format. One file collected multispectral sensor data, and another the values collected by the LiDAR of each angle in the same passage.

**Figure 1:**
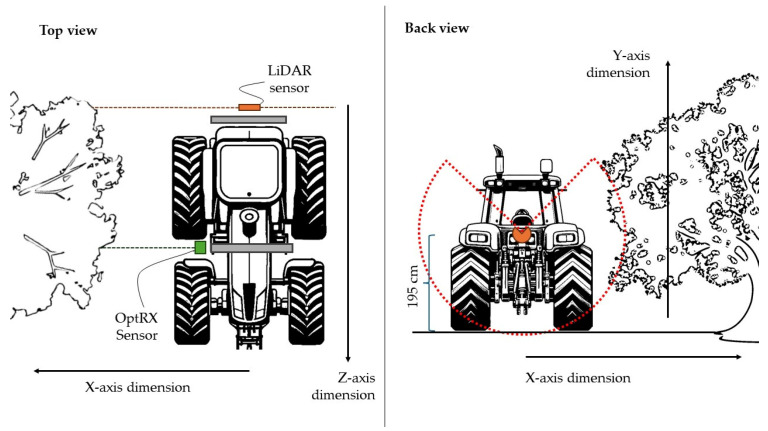
Schematic representation of data collection. The image represents the top view and the rear view of the tractor. In the top view, the LiDAR and OptRX sensors are shown in orange and green. The dotted lines indicate the path of the light beams emitted by the two sensors. In the case of the multispectral sensor, the emitted light beam can only monitor one side of the olive tree row; in the case of the 2D LiDAR, both sides can be detected. The rear view shows that LiDAR collects data with a scan angle of 270°. Light beams are emitted every 1/3 polar degree. In both images, the Cartesian axis of the data collected by LiDAR is specified.

For the MLS cloud point reconstruction, it was necessary to install a reference point to pair the two sides of the canopies. Four reference points, consisting of a 1 m height pole topped with a 0.2 × 0.2 m wood square, were distributed following the irrigation pipeline, between one plant and another, in the center of the rows. The two sensors, paired with the GNSS receiver, collected and recorded data every 0.2 m while the tractor maintained an average speed of 1.38 m s^−1^. Consequently, the points collected for each plant varied based on canopy length. Data were collected in a continuum, stopping at the end of each row. Figure 1 illustrates the different data collection methods and the positioning of the two sensors on the tractor. The Cartesian reference axes are also highlighted in the image: z for the tractor’s linear movement along the row, y for plant height, and x for plant thickness. These axes are fundamental in the LiDAR data processing, as will be explained below in Section 2.4.

### 2.3. Pruning Assessment

The ten plants selected for the case study were pruned in March 2023 following the farm pruning management, i.e., removing branches too protruding in the row, the branch, and the vigorous shoot and dry branches. The pruning was effectuated between the plant’s dormancy and the first bud break. The farm pruned the olive in turns of two years, and for experimentation, plants were not pruned the year before, which led to a visually dense canopy structure. To evaluate the magnitude of pruning and the digital biometrics measurements, the pruning was subdivided according to the exposition of the plants, i.e., the branches that started on the west side of the canopy were considered part of that side, and the branches that began on the east side were considered inherent to that side. The pruning operation was performed by the same operator for replicability.

After the operation, the pruning waste was weighed with the hanging scale UWE HS-30K (UWE EUROPE, Geldermalsen, The Netherlands). The two parts were weighed separately, and then the weight of the two parts was summed to obtain the total removed biomass weight.

### 2.4. Volume Assessment

**Figure 2:**
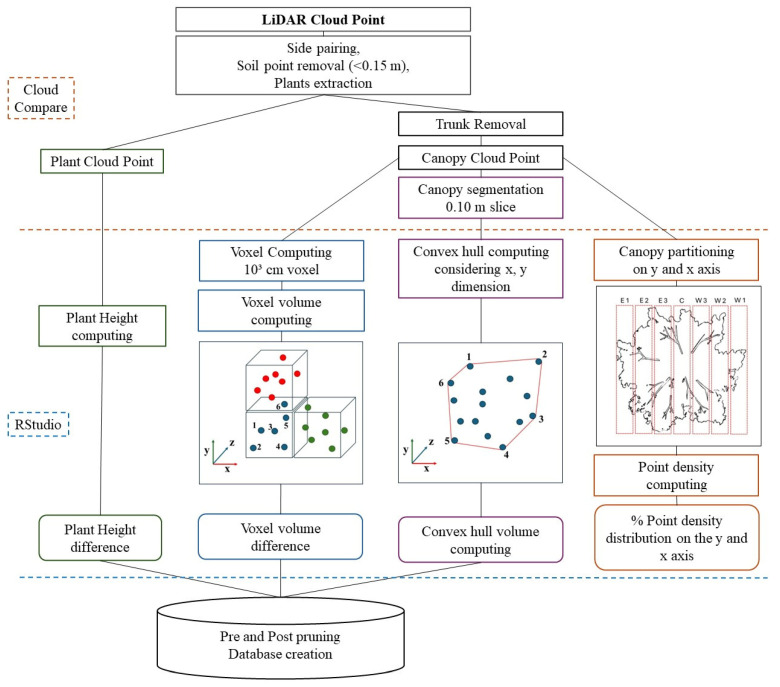
Workflow of the LiDAR data analysis. On the left side, it is possible to see the software (RStudio version 2023.06.0 Build 421, and Cloud Compare version 2.12.4-Kyiv, Ukraine) used in the various steps.

The LiDAR data were registered as the distance between the sensor and the first object that the light beam emits for all the different degrees. Consequently, the points were expressed in polar coordinates. With RStudio (version 2023.06.0 Build 421), the data were processed through trigonometry computation, deriving from the polar coordinates the Cartesian coordinates expressed as x, y, and z in an absolute reference system. The *z*-axis indicates the tractor path and, consequently, the plant length as seen from the row; the *y*-axis represents the plant height, and the *x*-axis represents the plant thickness as seen from the inter-row. Cloud Compare software (EDF, version 2.12.4-Kyiv, Ukraine) further processed the new coordinates representing the point cloud. In the Cloud Compare space, it was possible to reconstruct the plant crowns. Since the 2D LiDAR used in the experimentation can collect points linearly on just one side of the row at a time, the two sides of the row must be paired to reconstruct the plant. The reference points described in Section 2.2 were used to ensure the canopies were reconstructed correctly; the reference points were positioned in the center of the inter-row and gave the position reference of the midpoint of the canopies. Consequently, the LiDAR also recorded the point of these references, creating an intermediate reference used to crop the point cloud correctly. After the cropping, the two sides were coupled, creating a unique cloud point for each row. After this preliminary reconstruction, a cloud point of the olives rows was obtained. But those cloud points still contained the point representing the soil; consequently, a cleaning was needed to obtain the cloud point of the plants only. The soil points were cropped out of the cloud point using the CANUPO terrain classifiers algorithm in Cloud Compare. Two classifiers were chosen, one for the soil point cloud and one for the plants point cloud. Training points to instruct the algorithm were classified as soils (identifier 1) and plants (identifier 2) and were used to segment the point cloud. After that, the single sampled plants were extrapolated from the entire rows, and a separate cloud point was created for each plant. Finally, the trunk points were cropped out from the plants’ cloud points. In this case, the CANUPO segmentation algorithm, built into Cloud Compare, was trained to classify the canopies (identifier 1) and the trunks (identifier 2) into two sections. This way, two different point clouds were obtained: one representing the entire plant and one representing the crown alone. The point clouds were further analyzed in RStudio using the *VoxR* package [48], and it was possible to measure the maximum plant height (indicated as Height Plant from LiDAR–HPL) using the point cloud of the plant. Using the point cloud of the canopy instead, the total height of the canopy alone (identified as Height Canopy from LiDAR–HCL) was measured. Subtracting the dimension of the total plant height and the canopy height, it was possible to calculate the trunk height (identified as Height Trunk from LiDAR–HTL). The diameter of the canopies obtained by the point clouds (Diameter of the Canopy from LiDAR-DCL) was also estimated. The point clouds of the canopy were further analyzed and used to calculate the apparent volume of the plants. The term apparent volume is preferred because both methods used to calculate the volume with MLS measurement—convex hull and voxel system—can provide an estimate that may be subject to some error [49]. Figure 2 summarizes the workflow used for managing the LiDAR cloud point data for the different elaborations. 

Table 1 summarizes the different systems used for the biometric measurement estimation.

#### 2.4.1. Manual Measures

Manual measurements were taken for each sampled plant before and after pruning. The measurements were used as an element of control for the MLS canopy reconstruction. The measurements were taken with red and white survey poles, with a different color every 0.2 m. Maximum canopy diameter (identified as Diameter Canopy from Manual measurement-DCM), maximum plant height (identified as Height Plant from Manual measurements-HPM), total canopy height (identified as Height Canopy from Manual measurements-HCM), and plant trunk height (identified as Height Trunk from Manual measurement-HTM) were measured three times for repetition. The average of the three measurements was used as the height and diameter value representative for each plant. With those measurements, plant volume before and after pruning was calculated. The volume was calculated using two different systems. The first consisted of simplifying the canopy shape as a cylinder (VCY), i.e., it was considered that the whole canopy could be inscribed inside a geometric cylinder. To compute the volume, the cylinder volume formula (cylinder volume = π × cylinder radius^2^ × cylinder height) was used. The diameter and the height measured manually were used for the cylinder volume computation. The second system consisted of simplifying the canopy shape as a sphere (VSH), i.e., it was considered simplification as all the canopy could fit inside a geometric sphere. To compute the volumes, the sphere volume formula was used (sphere volume = (4/3) × π × sphere radious^3^). In the sphere volume computing, the radius was obtained as a mean between all diameter and height measurements taken for each plant divided by two. 

#### 2.4.2. Convex Hull Volume Estimation

For the volume estimation using the convex hull algorithm (VCH), it was necessary to split the canopies’ cloud points into slices. To do so, the canopies’ point clouds were segmented in slices 0.1 m wide through the Cross Section Segmentation function in Cloud Compare. The point clouds were segmented following the plants’ width, creating multiple sections for each plant. In RStudio, the segments were further elaborated to obtain the convex hull area. For each of these segments, the convex hull algorithm was used to estimate the smallest area that included all the points of the cloud following a process similar to the one proposed by Miranda-Fuentes et al. [50]. The *grDevices* package was used to create the convex hull [51]. For every segment created, the area of the convex hull was computed, which was subsequently multiplied by the thickness of the segment itself, as explained in function 3.

(3) $$V_{i}=\sum_{1}^{n}\Delta w\;\times \;A_{c,i}$$

where *V_i_* is the apparent volume of *i* plant, Δ*w* is the width difference, and *A_c,i_* is the area of every convex hull belonging to *i*.

The system can compute the apparent volume of the canopies. In this case, the potential error is because the convex hull algorithm does not consider the potential empty internal part of the canopy, leading to potentially wider area and volume results than reality.

#### 2.4.3. Voxel Volume Estimation

Voxel apparent volume estimation (VV) was performed to obtain a second way of computing the volumes from point clouds [49,52,53]. This estimation system creates voxels (cubes) of identical dimensions and volume. The point clouds were elaborated in RStudio with the *VoxR* package. The voxelization was set up to have voxels of predefined size and to have at least one point of the point cloud inside each voxel. The volume was estimated by multiplying the number of voxels with at least one point inside it by the volume of the voxels themselves, as explained in Equation (4).

(4) $$V_{i}=N_{i}\times v$$

where *V_i_* is the apparent volume of *i* plant, *N_i_* is the number of voxels belonging to *i*, and *v* is the volume of a single voxel.

Every single voxel created was 10^−5^ m^3^ in volume, as this dimension was observed to be more representative of the canopy’s volume and structure compared to the volume results of voxelization made with smaller or bigger voxels. With a smaller voxel, the risk of errors in the estimation due to the voxel containing just one point led to an overestimation of the volumes. In the case of bigger voxels, the complexity of the canopy shapes was simplified, causing an error in the estimation of the volumes. Consequently, the dimension of the voxel can lead to error in overestimation or lower estimation. The dimensions set for voxelization were those that allowed us to minimize those errors.

The system can compute the apparent volume of the canopies: in this case, it was possible to evaluate the potential empty internal part of the canopy.

### 2.5. Point Density Estimation

In addition to the biometric characteristics of the plants, a study was conducted on the density of the point cloud before and after pruning. The point density was defined as the number of impacts of the LiDAR beam on the target in a cubic meter. An approach similar to [54] was used to establish the point density assessment methodology. To evaluate the density of the number of impacts in a known volume, the point clouds were divided into sections—or “bins”.

Two dimensions were explored with this system, the *y*-axis and the *x*-axis. The *y*-axis, i.e., the height of the plant, was of interest to identify not only the different heights between before and after treatment but also to see potential differences in the vertical profile of the plant. The *x*-axis (i.e., the thickness of the plant) was studied to assess whether the internal and external profile of the plant registered different point cloud density values after the pruning.

Different methodologies were used to create the bins subdividing the canopy point cloud for the point density measurement. In the case of the *y*-axis, it was decided to divide the crown into equal sections. Bins of 0.4 × 0.4 × 0.4 m were therefore created. Those bins subdivided the *y*-axis into several subsections. The number of the section depended on the total height of the canopy. For each height section, *n* bins of equal size were obtained. For each bin, the point density was calculated. To obtain the point density, the number of points contained inside each bin was computed by using a summarizing function in Rstudio to compute the group size (*dplyr* package [55]). The points counted were then divided by the volume of the bins, obtaining the point density expressed as the total number of points present in the volume of the bins, i.e., n° points × m^−3^. The mean density of the *n* bins within each section of the *y*-axis was calculated. This approach was chosen to obtain results that also included the evolution of the horizontal density distribution of the point cloud of the canopy.

Considering the *x*-axis, it was necessary to create seven bins to partition the point cloud (one in the center and three for each exposition). Equation (5) summarizes the calculation for the size of the bins and the sequencing of the cloud points on the *x*-axis.

(5) $$Wb={[(max}_{x}-{min}_{x})/2]/a\;{Sec}_{n}^{1}=\mu \;\pm a*Wb$$

where *Wb* is the bin width, *a* is the value necessary to obtain the correct number of bins to sect the canopy in the *x*-axis. In this case, *a* was equal to 3.5, obtaining 7 sections. *μ* is the mid-point of the canopy. ${Sec}_{n}^{1}$ is the regular sequence in which the *x*-axis will be partitioned. The z and y dimensions for the bins volume computation were computed as the total height and length of the specific bin; consequently, the canopies point clouds were subdivided into 7 sections. The seven sections were named according to the position and the exposition: E for east, W for west, C for the center, 1 for the outside slices, 2 for the middle, and 3 for the innermost.

The point density was computed as in the *y*-axis case.

**Figure 3:**
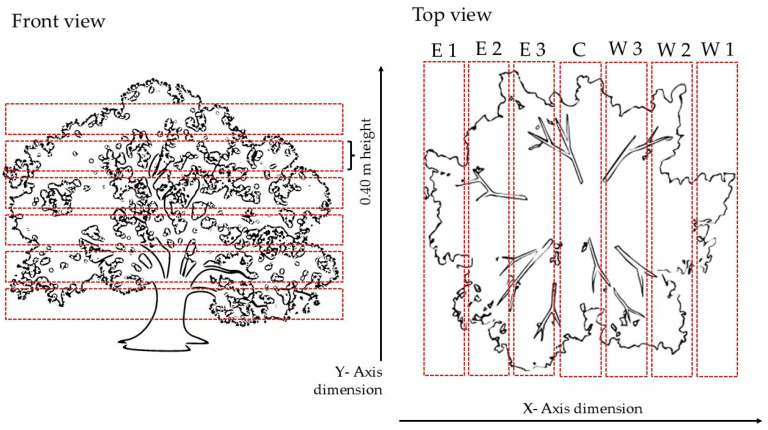
Plant *x*- and *y*-axis partitioning for point density estimation at different depths and heights of the plant. The front view indicates the view of the plant from the row. In this case, the point cloud data refers to the *y*-axis analysis, representing the height of the canopy, and the point cloud data were partitioned every 0.4 m. The top view of the canopy indicates the view from above. The letters state the position based on the exposition (E for east, W for west, and C for the center) and the number of the position based on the canopy thickness (outside slices, two for the middle, and three for the innermost). In this case, the data referred to the thickness of the plant, and the dimension of the section varied according to the total thickness of the canopy.

Figure 3 explains graphically the subdivision of the plants and the distribution of the bins created.

The point density distribution in the bins, for the *x*-axis, was then calculated as a point density percentage distribution. This information was considered of interest to assess the different distribution of the points in the point cloud before and after the pruning, i.e., to compare the point distribution in the canopies dimensions pre- and post-pruning, it was deemed necessary to evaluate not only the difference in the density results in the bins but also the overall distribution of the points in the bins. In particular, the objective was to assess the difference in the density of points in the area inside the canopy and whether pruning affected this value. Since the size of the sectors varied as the diameter of the canopy varied, in this case, it was preferred to express the density values as a percentage of the density in a sector out of the total measured density.

### 2.6. Statistical Analysis

VIs, WLs, volume, and pruning weight data were stored in Excel sheets (Microsoft CO, Washington, DC, USA). For VIs, cleaning the data from outliers or wrong values was necessary. The multispectral data were in point form, with each point characterized by its spatial location and the measured wavelength and VIs. Points with negative wavelength values corresponding to empty areas (i.e., measurements taken between plants) were considered erroneous, and values exceeding the VI threshold referring to vegetation (from 0 to +1) were considered outliers. After the cleaning, the multispectral sensor data were sampled through GIS software (QGis 3.18 Zürich, Switzerland). A dataset of 100 values of the two VIs was extracted for each sampled plant, separating the points collected to the East from the points collected from the West side of the plants, obtaining 200 points for each plant. After the sampling, statistical analysis was performed using RStudio. Tukey transformations were performed to normalize data for VIs and WLs data (for red edge, NDRE, and NDVI cases). The ANOVA analysis was performed to evaluate the effect of pruning on the response of WLs and VIs by comparing the data before and after the pruning (null hypothesis H_0_ = no difference between before and after the treatment). After the ANOVA, Tukey’s test for multiple comparisons was used to estimate the difference between the WLs and VIs, considering the effect of the pruning. Bonferroni confidence level correction was applied to Tukey’s test, and *p*-values were considered significant at α = 0.05. The “emmean” R package was used to apply the test.

A Pearson correlation method compared the manual and LiDAR methods of estimating volume, diameter, and height. Pearson coefficient values greater than 0.6 and with a *p* < 0.05 were considered significant. The difference between the estimated values of volume, canopy height, and canopy diameter before and after pruning was calculated by the previously described systems. The differences obtained were correlated with pruning weight. Pearson’s method was used to estimate the correlation, as previously explained.

Finally, a correlation study was performed, relating the VIs and WLs data to the canopy point cloud density data obtained, as explained in Section 2.5. The two sides of the plants (east exposure and west exposure) were considered separately for VIs, WLs, and point density. Pearson coefficient values greater than 0.6 and with a *p* < 0.05 were considered significant.

## 3. Results

### 3.1. Canopies Characterization

#### 3.1.1. Plant Measurement

The mean volumes, heights, and diameters of the ten plants sampled before and after pruning are presented in Table 2. For the volumes, data from the four estimation systems are presented, considering separately the LiDAR estimation systems and the manual estimation systems. It is possible to see that there was a decrease in volume values before and after pruning for all systems, although the differences are not statistically significant. Only in the case of VSP volume estimation was a statistical difference found between before and after treatment. It can also be observed that the various volume estimation systems lead to very different mean volume values, with the VCY system measuring the highest values (32.972 m^3^ and 23.318 m^3^) and the VV system measuring the lowest (7.242 m^3^ and 6.41 m^3^). Another difference between the estimation systems can be seen in the average volume differences between before and after pruning. The slightest difference was for VV (0.832 m^3^) and VCH (2.314 m^3^), and the largest in the case of VCY (9.609 m^3^) and VSP (6.378 m^3^).

After pruning, it was observed that all the samples measured a statistical decrease in total plant (HPL and HPM) and canopy (HCL and HCM) heights. This result is common to the two measurement methodologies used: LiDAR and manual. The trunk height (HTL and HTM) tended to be higher after pruning; however, the differences between before and after the treatment were not statistically significant. It was also possible to assess minor diameter results post-pruning compared to the results before the treatment.

Considering the different methodologies for estimating the canopy’s measurements, it was possible to assess that the LiDAR measurements tended to be higher than the manual estimation, particularly in the diameter cases. In this case, the LiDAR sensor recorded values up to 25% higher than those obtained with manual measurements.

**Figure 4:**
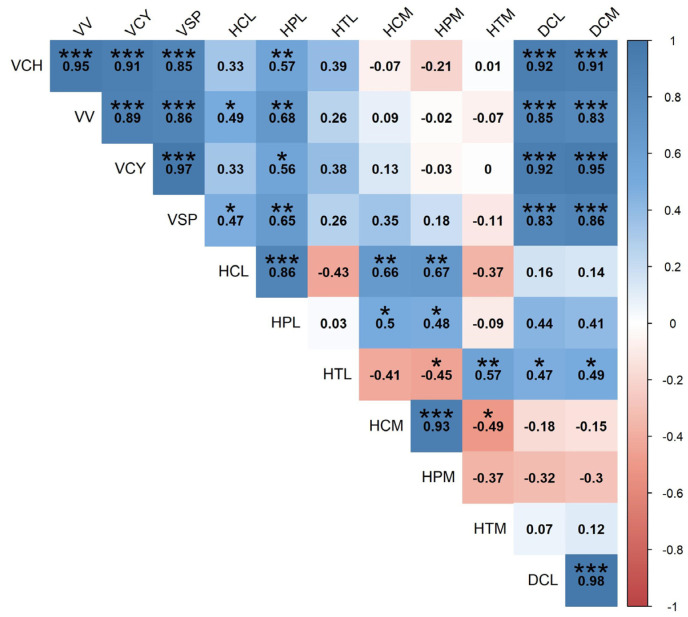
Correlation matrix between the multiple measurement systems for the canopy’s volumes, heights, and diameters. The numbers state the Pearson coefficient, and the colors assess the positive or negative values of the coefficient. The p values are expressed with the following symbols: *p* < 0.1 (*), *p* < 0.05 (**), and *p* < 0.01 (***).

The correlation matrix in Figure 4 shows correlation analysis between the different biometric measurement systems. The volume estimation systems based on LiDAR data (VV and VCH) correlated almost perfectly with each other (r = 0.95). Volume estimation based on manual measurements (VSP and VCY) also achieved a high level of correlation with the volume obtained from LiDAR. Furthermore, canopy volumes correlated better with canopy diameter than with canopy height.

The height measurements give mixed results; HCL correlates well with HCM and HPM, while HPL correlates poorly with the two manual measurements. Nonetheless, even if the correlation values between the different height measurement systems were low (0.66–0.67), the values obtained allowed us to establish good correlations between the methodologies. Furthermore, HTL correlates poorly with HTM, indicating a substantial difference in the performance of the two variables, as also shown in Table 2. HTL and HTM both have a negative correlation with the corresponding HCL and HCM, although only the latter is statistically significant. This follows at least in part the trend seen in Table 2. The diameter measurement obtained with the two methodologies (DCM and DCL) correlates perfectly.

#### 3.1.2. Pruning Assessment Results

Table 3 shows the mean and standard deviation weight of pruning for each plant pruned. The data in the table shows a fair variability between samples in the total weight of wood removed by pruning. On average, more than 9 kg of wood was removed from the selected plants. Considering the two expositions separately, it can be observed that the weight of pruning in the west-facing area was higher than in the east-facing area.

### 3.2. Cloud Point Density Study

**Figure 5:**
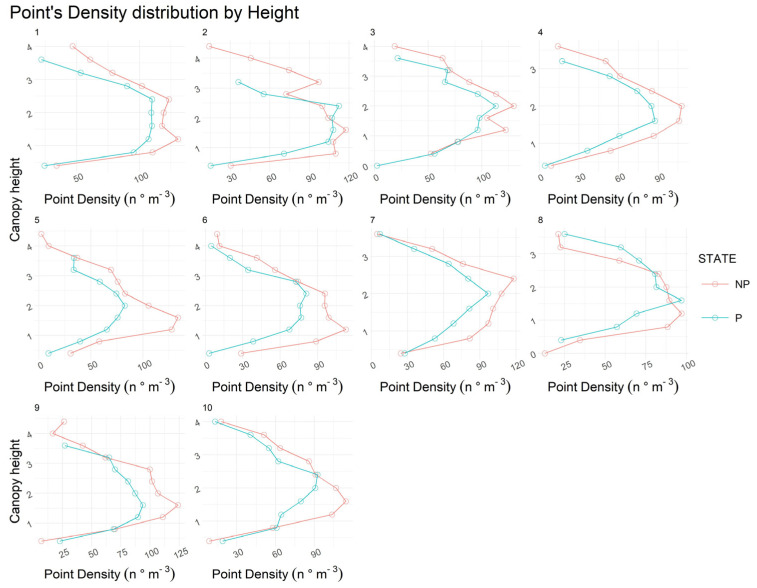
The graphs show the different point densities at various heights before and after the pruning for the ten sampled plants. The point density is expressed as the density of points per volume in m^3^ (n° *point* × m^−3^), and the canopy height is m. The red lines and dots are the values before the pruning, and the blue lines and dots are the values after the pruning.

Point cloud density was analyzed separately, considering plant height (*y*-axis) and plant thickness (*x*-axis). This method allowed the observation of the variations in the density of the point cloud, expressed as the number of points (LiDAR impact) on m^3^, before and after pruning, focusing especially on identifying the variation in the distribution of the point cloud on the two axes considered. In the height-related study, the canopies were subdivided every 0.4 m, making it possible to show the differences in canopy height alone between before and after pruning. The results are shown in Figure 5. Different shapes and patterns of the curve created by the different densities at various heights are evident.

Before pruning, LiDAR obtained plants with more jagged and irregular crowns with overhanging branches, especially in the lower part of the crown. After pruning, on the other hand, the shape of the curve was more regular, showing that the canopy had taken on a more regular shape, reducing the length of the branches protruding toward the inter-row. This is particularly evident in plants 2, 5, and 9, where the canopies presented a more compact and regular shape after the pruning, with a more concentrated density in the middle part of the canopy at 2 m. It is also noted that point densities after pruning were lower than before treatment, but these differences are not statistically significant.

**Figure 6:**
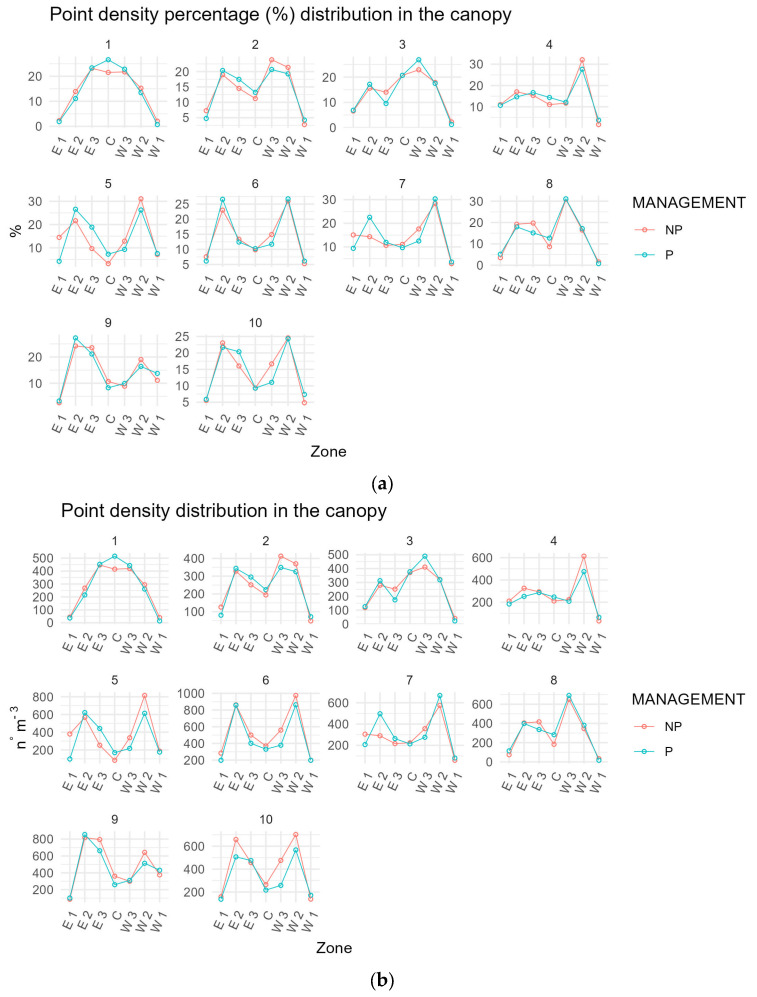
Graph (**a**) show the difference in the percent distribution of point density for the ten trees sampled in the seven plant sections. Graph (**b**) show the distribution of point density for the ten trees sampled in the seven plant sections where C is the middle section, E is the section in the east exposure, W is the section in the west exposure, and 1, 2, and 3 are the outer, middle, and inner sections. Red dots and lines refer to unpruned values; blue dots and lines refer to pruned values.

The graph a in Figure 6 show that the percentage distributions of values remain almost unchanged between before and after pruning for nearly all plants. Minimal differences occur in the case of plant 5 and plant 2. As for the central area of the plant, i.e., sector C, in five cases, this shows a lower percentage of density distribution after pruning than in the initial condition. In two cases, higher values are registered after the pruning, and in the remaining three, they are unchanged. Moreover, the west-facing side of the plants tends to record a higher density than the east-facing side; this condition is evident for plants 3, 4, 5, 7, 8, and 10. A similar pattern can be seen in the density distribution shown in graph b.

### 3.3. Pruned Weight Correlation Study

The results of the correlation study between the weights of woody matter removed at pruning and the differences in volume before and after pruning are shown in Table 4. It can be seen that all correlations have high r (Pearson’s correlation coefficient) values and are substantiated by statistical significance. Therefore, all systems, including those based on the LiDAR system, can potentially appropriately determine the weight removed from the plant at pruning. It can be seen that the highest correlation values were recorded in the case of manual measurements. In contrast, in the case of measurements from LiDAR, r values are lower, but they remain valid for estimating pruning wood removal.

### 3.4. Relationship Between VIs, WLs, and Cloud Point Density

#### 3.4.1. VIs and WLs Measurements

Results of VIs and WLs before (not pruned, NP) and after (pruned, P) pruning are shown in Table 5. The results are expressed as the mean and standard deviation of 200 sampled points per plant (100 in the west-facing part of the canopy and 100 in the east-facing part). The difference in the results between the two expositions was studied through an ANOVA and Tukey’s multiple comparison test. No statistically significant differences were obtained; consequently, the values of the VIs and WLs are presented without considering the exposition. Different letters at the sides of the values indicate a statistical difference between the variables before and after pruning. It is possible to see that there are no evident differences between treatments, except for the red edge wavelength and the NDRE vegetative index. Even in these cases, the differences remain minimal. It is possible to note that there is a small decrease in the NIR values and an increase in the red and red edge values between before and after pruning, resulting in a consequent decrease in the values of the two VIs. The standard deviation values for every variable are smaller compared to pre-pruning values. Consequently, the values of the pruned plant present fewer variable results.

#### 3.4.2. Density, WL, and VI Correlation Studies

Table 6 and Table 7 show the correlation between the apparent density of the canopy (expressed as the density of the point cloud in m^3^) and the corresponding VIs and WLs before and after the pruning operation. The apparent density is referred to the data exposed in Section 3.2, particularly to the external part of the canopy (sum of the 1 and 2 sections). Considering the wavelength, before the pruning only the red wavelength showed a significant negative correlation with the point density, even if the Pearson value is lower than 0.6. NIR and red edge registered correlation values close to zero. After the pruning, the red WL obtained a significant negative correlation value with the point density equal to 0.62, an increase compared to before the operation.

In the cases of the WLs NIR and red edge, higher values were obtained after pruning. Nevertheless, these variations are to be considered insubstantial. NDRE registered, before the pruning, a positive but not significant correlation, slightly increased in value after the pruning, even if it is still not significant. NDVI, on the contrary, obtained a positive correlation with the point density, which resulted in a significant correlation after the pruning.

It can be observed that red and NDVI are the two parameters with the highest correlations (namely negative and positive) with point density. In contrast, red edge and NDRE show a poor relationship with canopy density. Both vegetative indices show an increasing trend and a positive relationship with density; on the contrary, the two wavelengths assess a decreasing trend and a negative relationship with canopy density.

## 4. Discussion

In this study, the effectiveness of a LiDAR and a proximal multispectral sensor in discerning variations in the canopy structure and vegetative behavior of olive trees pre- and post-pruning was assessed.

The use of the GMV’s LiDAR to estimate the volume, height, and diameter of the canopy provided values that correlated with the manual measurement, allowing us to state with a fair degree of certainty that the LiDAR estimate agrees well with the manual estimate, as also noted by previous studies [50,56,57]. These sensors, by allowing automation of data collection—particularly in a GMV terrestrial LiDAR—can speed up this process. Although the calculation of volumes still needs simplifications, particularly in the data processing part, the total information it allows us to collect is sufficiently detailed. Moreover, the critical feature of such a sensor is that it is potentially automated, allowing the obtaining of real-time data for field management. Nonetheless, the difference in the results for the various volume estimation systems can be problematic. The diameter and height values obtained from LiDAR were higher than the manual counterparts. The discrepancy between the measurements may be due to LiDAR’s higher sensitivity to include smaller elements—leaves or branches—in the point cloud (and consequently in the estimation of biometric parameters). Such elements can potentially be excluded in manual measurements that, even if optimally performed, always contain an element of uncertainty due to the perceptual limitation of the human eye [50]. The volume estimations registered very high values in m^3^, ranging from 32 to 18 m^3^. Those are similar to previous studies [26,34,50,58], but compared to VCH, VSH, and VCY, the VV values record much smaller ranges of volume. The difference between the three other systems and VV was up to 25 m^3^, a consistent discrepancy. It can be assumed that the Voxel methodology performs differently because it ignores the hollow areas of the canopy. Cubic voxels are distributed into the point cloud in a way that allows them to contain at least one point. This distribution causes the voxels to follow the shapes of the plant, branches, and leaves that the LiDAR beam impacted. This results in a more detailed and less simplified representation of the volume, since by following the shapes and connotations detected by the sensor in detail, it is assumed that a volumetric computation can be obtained that is subject to less simplification and can better represent the complexity of the plant structure, as other studies, focusing on the relationship between LAI and LiDAR point clouds, stated [59]. This element needs further study, as it would strongly influence the volumetry results obtained with a LiDAR sensor mounted on a GMV. Consequently, this system still needs some evaluation and improvement [60], particularly if high precision in estimation is required. Nonetheless, on the results obtained, the authors estimate more valuable the application of the Voxel measurements method in the estimation of the volumes, as it can lead to more precise assessment and is capable of accounting for the complexity of the canopy geometries. Another element to consider is the time needed to perform the estimation: VCH was the most time-consuming analysis, as it required additional elaborations compared to VV. The two manual estimation methods were the least time-consuming but also led to the most approximate volume estimations. Moreover, the VV estimation correlated better with the pruning weight compared with the VCH methods; consequently, VV can give more correct predictions on the removed biomass after the pruning. 

Considering the pruning operation, the LiDAR sensor provided measurements of the canopies and plants’ height, which is functional for deducting the difference in the development after the pruning. In particular, the results showed a reduced plant and canopy height after the pruning, and a higher trunk height makes it possible to state that the crowns were shorter after pruning due to both the reduction in overall height and the increase in trunk height. The trunk height differences show that the pruning also removed woody material from the more basal parts of the crown, thus exposing the trunk more. The differences are especially notable in the cases of intense pruning operations that remove a substantial amount of wood. Still, limitations of the information provided by the LiDAR are present, as from the results it was not possible to assess if an actual difference occurred inside the canopy after pruning. Considering that usually the interior part of the canopy in a traditional olive orchard is left hollow after the pruning, allowing the light to penetrate inside the structure [61], it was expected to highlight some differences in the LiDAR impacts in the innermost part of the canopies. Considering that was not the case, it can be assumed that with a very thick canopy, the LiDAR cannot penetrate the inside of the canopy itself sufficiently; consequently, it cannot detect with a reasonable degree of certainty if a variation in the deepest part of the canopy occurred after the pruning, potentially overlooking important information for canopy management and other operations [15]. Nonetheless, the results presented allowed us to state that using GMV LiDAR data to detect the total pruned wood removed is efficient and can be potentially used for data collection for precision management, decision-making, and the creation of prediction models for estimating the total amount of removed wood from an orchard, particularly to assess the vegetative and productive balance [27,62]. Other elements in this regard are relatives to the canopy shape estimate that can be obtained with the LiDAR data. As the results in Figure 5 show, before and after pruning, the shape of the canopy changed, obtaining a more regular shape after the pruning. This element can be of interest for future canopy management operations, mechanical pruning, and automatization [42].

The effect of the pruning on the response to the two vegetation indexes investigated in this study—NDVI and NDRE—is still yet to be determined. It was possible to assess a statistical difference in the results after the pruning in the red edge and, consequently, NDRE values. However, the other variables, even if they registered a slight decrease in the values, were not statistically different before and after the treatments. This could be explained by the different responses to the biomass density of the different WLs. In particular, the NDVI index is known to be sensitive to high-biomass canopies, particularly in canopies with high values of LAI [63,64], as its relationship with LAI is exponential [65]. This condition is not present in the case of the NDRE [66]: the shape and slope of the reflectance curve in the red edge wavelength region, which is used to compute the NDRE index, is strongly influenced by LAI [67]. Consequently, the NDRE index is supposed to be more sensitive to biomass variation caused by pruning. It is essential to highlight, nonetheless, that even if the NDRE and red edge results were statistically different before and after the pruning, this difference is still low and cannot lead to total certainty of the effect of the pruning on the behavior of those VI and WL. It can be assumed that multispectral sensors provided wavelengths and indices may not be sufficient to estimate a change in biomass due to pruning. As other studies have suggested [68], it may be necessary to apply indices using narrow bands or bands more closely localized in the red edge region to estimate biomass changes more effectively.

The point density was correlated with the WLs and the VIs to evaluate whether the changes in the apparent density of the plant detected by the LiDAR could give information on the VIs’ and WLs’ potential behavior. This information was considered helpful for pruning management to obtain a correct equilibrium between the canopy density, the potential removed wood, and the plant’s vigor. The study resulted in an absence of relationships, highlighted by the low Pearson values, for almost all the variables. Nonetheless, for every studied WL and VI, the relationship was more robust after the pruning, with higher correlation values, in particular in the red and red edge cases, stating a potential influence of the apparent density in the response of the vegetation index.

Another element to consider is the low number of samples. It should be noted that even if ten plants were sufficient to obtain good results, enhancing the number of replicas can provide stronger results. Future studies must consider a larger sample population to confirm the trend. Another element to consider is the variability of the canopy characteristics depending on the cultivar. As olive trees have different canopy densities and behavior habits depending on the cultivar, it cannot be affirmed that the results obtained in this case study can be representative of all olive cultivars. In addition, this case study was carried out in a traditional olive grove; consequently, there is the possibility of obtaining different results in different management systems, especially considering those with continuous and denser vegetation, such as hyper-intensive olive groves [69]. 

## 5. Conclusions

This case study aimed to assess whether pruning and, therefore, the removal of wood from olive trees can be detected by two sensors: a multispectral sensor capable of discerning the vegetative vigor of the plants and a LiDAR sensor that, by creating a point, can assess the volume and shape of the plant. What has been observed is that the results obtained by both sensors appear to be influenced by pruning.

The LiDAR sensor showed differences between before and after pruning in terms of the difference in volume and the difference in the plant’s shape and its characteristics, such as height and diameter. These results allow us to affirm that the sensor can be a good tool for decision-making and management of the olive grove, as it will enable us to have predictive elements relative to the pruning weight removed, in addition to the fact that it can give volumetric information relative to an entire orchard with a time-saving in terms of data collection compared to manual measurement techniques.

With regard to the multispectral sensor, on the other hand, it was observed that its results are relatively unaffected by changes in canopy conformation due to pruning. Consequently, the application of this sensor in different conditions of canopy density can give stable information. The density of the point cloud collected with the LiDAR and the correlated multispectral sensor results, as analyzed from the perspective of this research, do not interact significantly with each other. It was possible to see a difference between before and after pruning in the relationship between the parameters. This variation does not allow us to state with absolute certainty that no interaction between the parameters is possible, and consequently, this interaction must be further studied. From the results, however, it can be deduced that the joint data collection of point density and spectral index response may not lead to agronomic and operational interest information. In conclusion, the two sensors gave different responses to the canopy changes, as the multispectral sensor was not affected by the treatment; instead, the LiDAR showed different results. This case study allowed an initial evaluation of the application of LiDAR and multispectral sensors as tools that can be used for data collection in pruning management, highlighting some weaknesses and the need for further research. Firstly, future studies should consider increasing the number of replications to confirm the results, especially those related to the relationship between canopy density from LiDAR and multispectral data, as it is assumed that the low number of samples may influence the relationship. Secondly, it is necessary to evaluate different forms of management and planting layouts, which may be crucial in assessing the effectiveness of LiDAR in detecting plant biometric parameters and biomass variation due to pruning, particularly in hyper-intensive olive groves. Thirdly, other olive cultivars need to be considered; in particular, future studies should focus on considering multiple replications of multiple olive cultivars to create a more robust and meaningful experimentation. Fourth, other volume computation systems should be explored, in particular for the automatization of the data collection and management. Finally, given the inconsistency of the vegetative indices’ response to pruning, it will be necessary to consider other sensors, particularly hyperspectral sensors that can provide more in-depth results in the red edge wavelength region, as well as other vegetative indices that are based on the red edge wavelength.

## Figures and Tables

**Table 1:** Different methods and instruments used for volume biometric measurements. The simplification method is volumetric computing based on the assumption that the entirety of the crown can be simplified in a geometric volume.

Biometric Measurement	Instrument	Method
Volume	LiDAR	Convex hull
	LiDAR	Voxelization
	Manual (graduated pole)	Sphere simplification
	Manual (graduated pole)	Cylinder simplification

**Table 2:** The means and standard deviation values relative to volume, height, and diameter assessment are exposed in the table. The volumes are expressed as m^3^, and the heights and diameters as m. Values before pruning are reported in the NP (not pruned) column, and data after pruning are in the P (pruned) column. Different letters indicate a statistically significant difference (*p* ≤ 0.05) in Tukey’s test. Each value for the parameters is the mean value of 10 samples.

Biometrics Measurements	NP	P
	µ ± σ	µ ± σ
VCH (m^3^)	18.484 ± 8.283 (a)	16.170 ± 6.775 (a)
VV (m^3^)	7.242 ± 1.947 (a)	6.41 ± 1.517 (a)
VCY(m^3^)	32.972 ± 12.06 (a)	23.318 ± 8.667 (a)
VSP(m^3^)	21.770 ± 5.971 (a)	15.392 ± 4.369 (b)
HCL (m)	3.512 ± 0.431 (a)	3.053 ± 0.226 (b)
HPL (m)	4.139 ± 0.408 (a)	3.791 ± 0.257 (b)
HTL (m)	0.627 ± 0. 25 (a)	0.737 ± 0.145 (a)
HCM (m)	3.41 ± 0.296 (a)	3.024 ± 0.289 (b)
HPM (m)	3.91 ± 0.299 (a)	3.594 ± 0.232 (b)
HTM (m)	0.50 ± 0.115 (a)	0.57 ± 0.139 (a)
DCL (m)	4.596 ± 0.860 (a)	4.272 ± 0.845 (a)
DCM (m)	3.47 ± 0.675 (a)	3.095 ± 0.647 (a)

**Table 3:** Mean and standard deviation of the pruned wood from each plant sampled.

	Total Weight (kg)	Weight East (kg)	Weight West (kg)
µ ± σ	9.373 ± 4632	8532 ± 4406	10,214 ± 4930

**Table 4:** Pearson correlation values between the pruned weight and the differences in volumes before and after the pruning for each of the four different volume estimation systems used: VCH (volume of the convex hull), VV (volume of the voxels), VCY (volume of the cylinder), and VSP (volume of the sphere). The p values are expressed with the following symbols: *p* < 0.05 (**), and *p* < 0.001 (***).

Differences	Weight
VCH	0.66 **
VV	0.74 **
VCY	0.82 ***
VSP	0.83 ***

**Table 5:** Mean and standard deviation for wavelengths and vegetative indices. Values before pruning are reported in the NP column, and data after pruning are in the P column. Letters next to the values indicate the presence or absence of statistical differences between the treatments. Different letters indicate a statistically significant difference (*p* ≤ 0.05) in Tukey’s test. Each parameter value is the mean and standard deviation of 200 samples.

	NP	P
	µ ± σ	µ ± σ
NIR	0.341 ± 0.07 (a)	0.339 ± 0.084 (a)
RED	0.077 ± 0.026 (a)	0.078 0.031 (a)
Red Edge	0.207 ± 0.014 (a)	0.210 ± 0.016 (b)
NDVI	0.629 ± 0.091 (a)	0.623 ± 0.096 (a)
NDRE	0.234 ± 0.101 (a)	0.226 ±0.110 (b)

**Table 6:** Correlation values between the WL, VI, and point density before the pruning. The *p*-values are expressed with the following symbols: *p* < 0.1 (*).

	Point Density
RED	−0.53 *
Red Edge	−0.12
NIR	−0.10
NDVI	0.40
NDRE	0.02

**Table 7:** Correlation values between the WL, VI, and point density after the pruning. The *p*-values are expressed with the following symbols: *p* < 0.05 (**).

	Point Density
RED	−0.62 **
Red Edge	−0.44
NIR	−0.19
NDVI	0.61 **
NDRE	0.34

## Data Availability

The dataset is available upon request from the authors.
